# Preoperative cervical sagittal alignment parameters and their impacts on myelopathy in patients with cervical spondylotic myelopathy: a retrospective study

**DOI:** 10.7717/peerj.4027

**Published:** 2017-11-09

**Authors:** Wei Yuan, Yue Zhu, Haitao Zhu, Cui Cui, Lei Pei, Zhuxi Huang

**Affiliations:** Department of Orthopedics, First Hospital of China Medical University, Shenyang, China

**Keywords:** Cervical lordosis, Sagittal vertical axis, Myelopathy, Cervical sagittal alignment, mJOA

## Abstract

**Background:**

Cervical sagittal alignment plays an important role in the pathogenesis of cervical spondylotic myelopathy (CSM), but there are limited studies on the cervical sagittal parameters in CSM patients and their correlations with myelopathy. The aim of this study is to investigate the correlations among the preoperative cervical sagittal alignment parameters and their correlations with the development of myelopathy in patients with CSM.

**Methods:**

We retrospectively collected 212 patients with CSM who underwent surgical interventions. Gender, age, modified Japanese Orthopedic Association score (mJOA), cervical lordosis (CL), C2–C7 sagittal vertical axis (C2–C7 SVA), T1 slope (T1S), neck tilt (NT) and thoracic inlet angle (TIA) were collected before operation. Interobserver and intraobserver reliability were calculated for all measurements (intraclass correlation coefficient, ICC). Data were analyzed with Pearson and Spearman correlation tests and multiple linear regression analysis.

**Results:**

A total of 212 patients with CSM were included in this study (male: 136, female: 76) with an average age of 54.5 ± 10.1 years old. Intraobserver and interobserver reliability for all included radiographic parameters presented good to excellent agreement (ICC > 0.7). No significant differences in demographic and radiological parameters have been observed between males and females (*P* > 0.05). We found statistically significant correlations among the following parameters: age with CL (*r* = 0.135, *P* = 0.049), age with T1S (*r* = 0.222, *P* = 0.001), CL with T1S (*r* = 0.291, *P* < 0.001), CL with C2-C7 SVA (*r* =  − 0.395, *P* < 0.001), mJOA with age (*r* =  − 0.274, *P* < 0.001), mJOA with C2–C7 SVA (*r* =  − 0.219, *P* < 0.001) and mJOA with *T*_1_*S*(*r* =  − 0.171, *p* = 0.013). Linear regression analysis showed that C2–C7 SVA was the predictor of CL (adjusted *R*^2^ = 0.152, *P* < 0.001) and multiple linear regression showed that age combined with C2–C7 SVA was a sensitive predictor of mJOA (adjusted *R*^2^ = 0.106, *P* < 0.001).

**Discussion:**

There were significant correlations among certain preoperative cervical sagittal parameters in CSM patients. CL was the only predictor of C2–C7 SVA. Age combined with C2–C7 SVA could predict the severity of myelopathy.

## Introduction

Cervical spondylotic myelopathy (CSM) is a degenerative disease and the most common cause of neurological dysfunctions in the world (Klineberg, 2010; Smith et al., 2013). A study of the CSM nature history indicates that 20%–62% of CSM patients without surgical interventions will present neurological deterioration three to six years within follow-up (Karadimas et al., 2013). Standard treatment of CSM often focuses on surgical decompression with an anterior or posterior approach (Tetreault, Karpova & Fehlings, 2015). Disc degeneration has been recognized as the initiating event of spondylotic changes, which can lead to abnormal cervical spine biomechanics and loss of normal sagittal alignment (Ferrara, 2012; Smith et al., 2013). Abnormalities of the cervical sagittal alignment, in return, could contribute to spinal cord dysfunction through several mechanisms, including direct compression, repeated flexion/extension injury and vascular compromise (Ames et al., 2013b; Smith et al., 2013). These findings highlight the importance of cervical sagittal alignment in the pathogenesis of CSM.

A number of studies have analyzed the cervical sagittal parameters in normal volunteers (Lee et al., 2012; Lee et al., 2015b; Yang et al., 2016), patients with idiopathic scoliosis (Youn et al., 2016), patients with ankylosing spondylitis (Lee et al., 2015a) and patients with cervical spondylolisthesis (Jun et al., 2015). The correlations among cervical lordosis (CL), C2–C7 sagittal vertical axis (C2–C7 SVA), T1 slope (T1S), neck tilt (NT) and thoracic inlet angle (TIA) also present varied results among studies. The interrelations among the cervical sagittal parameters in CSM patients and their correlations with myelopathy have not been fully elucidated (Smith et al., 2013; Tang et al., 2012; Weng et al., 2016). One study on cervical sagittal balance in degenerative cervical spine suggests that T1S has a positive correlation with CL (Weng et al., 2016). Instead of kyphosis, worse C2–C7 SVA was found to be correlated with greater myelopathy severity (Smith et al., 2013). The current study aims to investigate the preoperative cervical sagittal parameters in CSM, their intercorrelations and correlations between these parameters and myelopathy, which can demonstrate the clinical implications of cervical sagittal alignment in CSM patients.

## Materials and Methods

### Patient population

The inclusion criterion was patients with CSM that underwent surgical treatment in our department from January 2014 to August 2016. Exclusion criteria were: with previous history of cervical spine surgery, combined with congenital abnormality, history of hip or knee ankle surgery or any other realignment surgery of the lower extremities, complaints of low back pain and subjects without sufficient radiographic parameters. This study was approved by the Institutional Review Board of First Hospital of China Medical University (Number: 2014082), and all the patients fulfilled written informed consent for their information to be stored in the hospital database and used for research.

### Data collection

**Figure 1:**
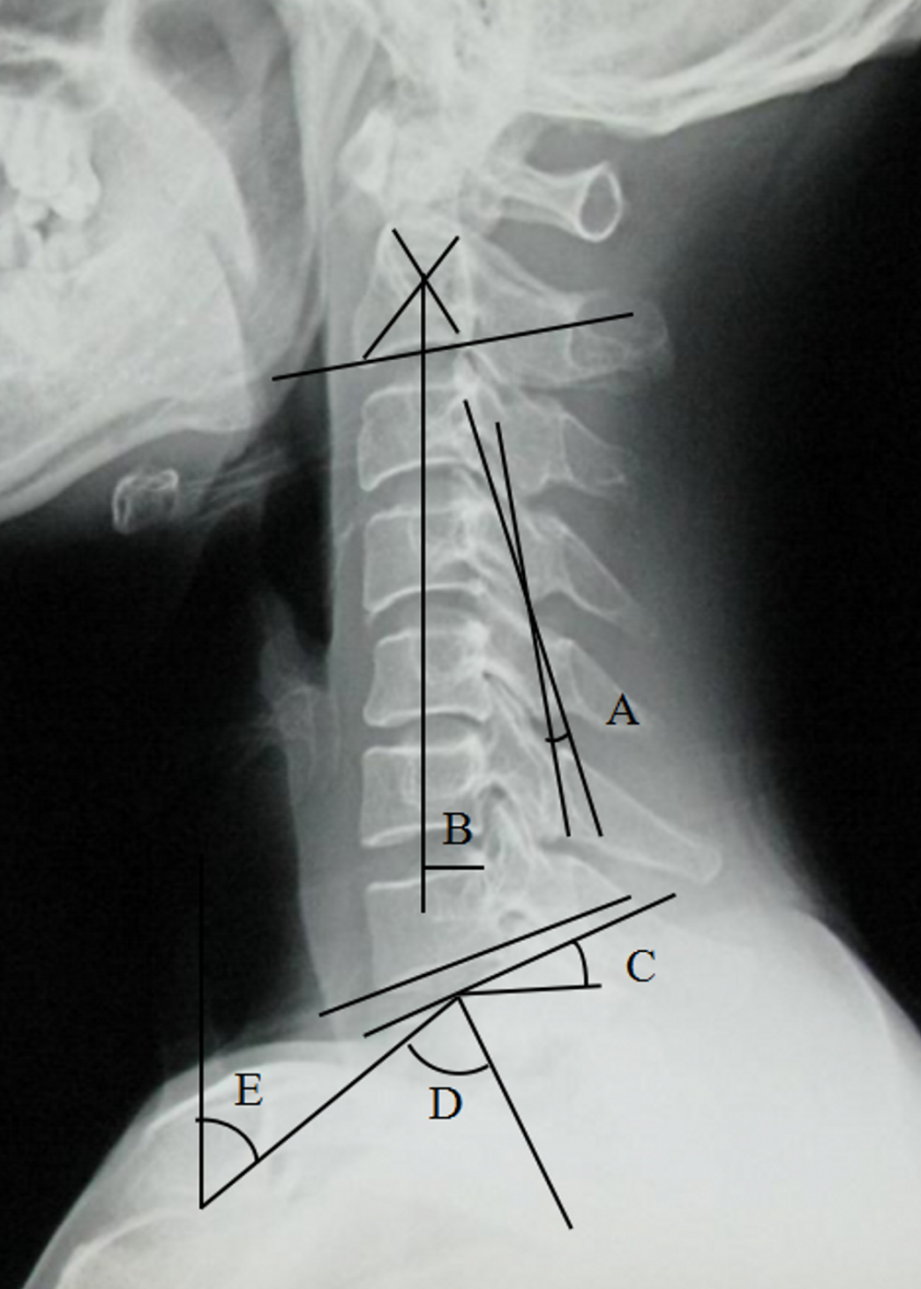
Preoperative radiographic measurement. (A) CL, (B) C2–7 SVA, (C) T1S, (D) TIA, (E) NT.

Demographic data including gender and age were collected. Preoperative modified Japanese Orthopedic Association score (mJOA) was extracted from the cases. The following cervical parameters were measured on the preoperative lateral radiographs (Fig. 1): CL, C2–C7 SVA, T1S, NT and TIA. CL was assessed by C2–C7 Cobb angle, which was defined as the angle formed by the inferior endplates of C2 and C7 in lateral radiographs. C2–C7 SVA was defined as the distance from the vertical line from the center of the C2 body and the posterior-superior corner of C7. T1S was defined as the angle formed between a horizontal line and the superior end plate of T1. NT was defined as the angle formed by the vertical line of the sternum tip and the line drawn in the center of the upper end plate of the sternum connecting the center of the T1 upper end plate. TIA was defined as the angle formed by a perpendicular line off the T1 upper end plate and another line connecting the center of the T1 upper end plate and the upper point of the sternum.

All measurements were performed twice independently by two spine surgeons with rich experience in radiographs measurement. The mean values were applied for analysis.

### Statistical analysis

Statistical analysis was performed using SPSS Ver. 20 for Windows (SPSS, Chicago, IL, USA). Descriptive statistics were expressed as mean ±  standard deviation. The independent sample *t* test was used to analyze the differences in the cervical spine parameters between males and females. Interobserver and intraobserver reliability was assessed using intraclass correlation coefficient (ICC). The internal consistency of the measurements was characterized as excellent (ICC  ≥ 0.9), good (0.7 < ICC < 0.9, acceptable (0.6 < ICC ≤ 0.7), poor (0.5 < ICC ≤ 0.6) and unpredictable (ICC ≤ 0.5) (Iyer et al., 2016). Correlation analysis was performed using Pearson and Spearman to study relationships between variables (gender, age, CL, T1S, NT, TIA, C2–C7 SVA and mJOA). Linear regression analysis was used to identify parameters that could predict C2–C7 SVA and mJOA. *P* values <0.05 were set as statistically significant.

## Results

A total of 212 patients with CSM (male: 136, female: 76) were recruited in our study with an average age of 54.5  ± 10.1 years old. Radiographic parameters for CSM patients in the current study and normal values reported in the literature (Iyer et al., 2016; Jun et al., 2014; Yokoyama et al., 2017) were listed in Table 1. There was no significant difference in terms of the parameters between CSM patients and normal controls (a value of more than one SD from the mean of reference normative values was considered significantly different) (Yoshida et al., 2017). In addition, no significant difference in terms of the demographic and radiological parameters was observed between males and females (*P* > 0.05) (Table 2).

**Table 1:** General characteristics and radiographic parameters in this study and normal values of asymptomatic subjects in literatures.

	CSM in this study	Normal subjects in Iyer et al., study	Normal subjects in Yokoyama et al., study	Normal subjects in Jun et al., study
Number of subjects	212	115	220	50
Gender, male (%)	136 (64.2)	36 (31.3)	99 (45)	30 (60)
Age (year)	54.5 ± 10.1	50.1 (22–78)	59.0 ± 17.4	47.84 ± 15.5
CL (°)	11.7 ± 11.1	12.2 ± 13.6	13.9 ± 14.2	17.3 ± 9.3
C2–C7 SVA (mm)	17.2 ± 12.1	21.3 ± 12.1	20.2 ± 11	–
T1S (°)	24.8 ± 10.5	26.1 ± 9	24.6 ± 7.5	25.97 ± 5.9
NT (°)	54.5 ± 12.3	51 ± 9.3	53.7 ± 10.2	48.7 ± 7.9
TIA (°)	79.4 ± 10.3	79.8 ± 13.3	79.5 ± 10.6	75.09 ± 8.1
mJOA	16.6 ± 4.7	–	–	–

**Notes.**

CSM cervical spondylotic myelopathy CL cervical lordosis C2–C7 SVA C2–C7 sagittal vertical axis T1S T1 slope NT neck tilt TIA thoracic inlet angle mJOA modified Japanese Orthopedic Association score

**Table 2:** General parameters in different genders.

	Male	Female	*P*
No.	136	76	
Age	54.2 ± 9.9	54.9 ± 10.5	0.641
CL (°)	12.6 ± 11	9.9 ± 11	0.98
C2–C7 SVA (mm)	17.8 ± 12	16.6 ± 10.8	0.459
T1S (°)	25.2 ± 9.9	24 ± 11.6	0.411
NT (°)	54.7 ± 11	54.1 ± 14.3	0.729
TIA (°)	80.1 ± 10.2	78.1 ± 10.3	0.17
mJOA	16.7 ± 4.7	16.6 ± 4.7	0.88

**Notes.**

CL cervical lordosis C2–C7 SVA C2–C7 sagittal vertical axis T1S T1 slope NT neck tilt TIA thoracic inlet angle mJOA modified Japanese Orthopedic Association score

**Figure 2:**
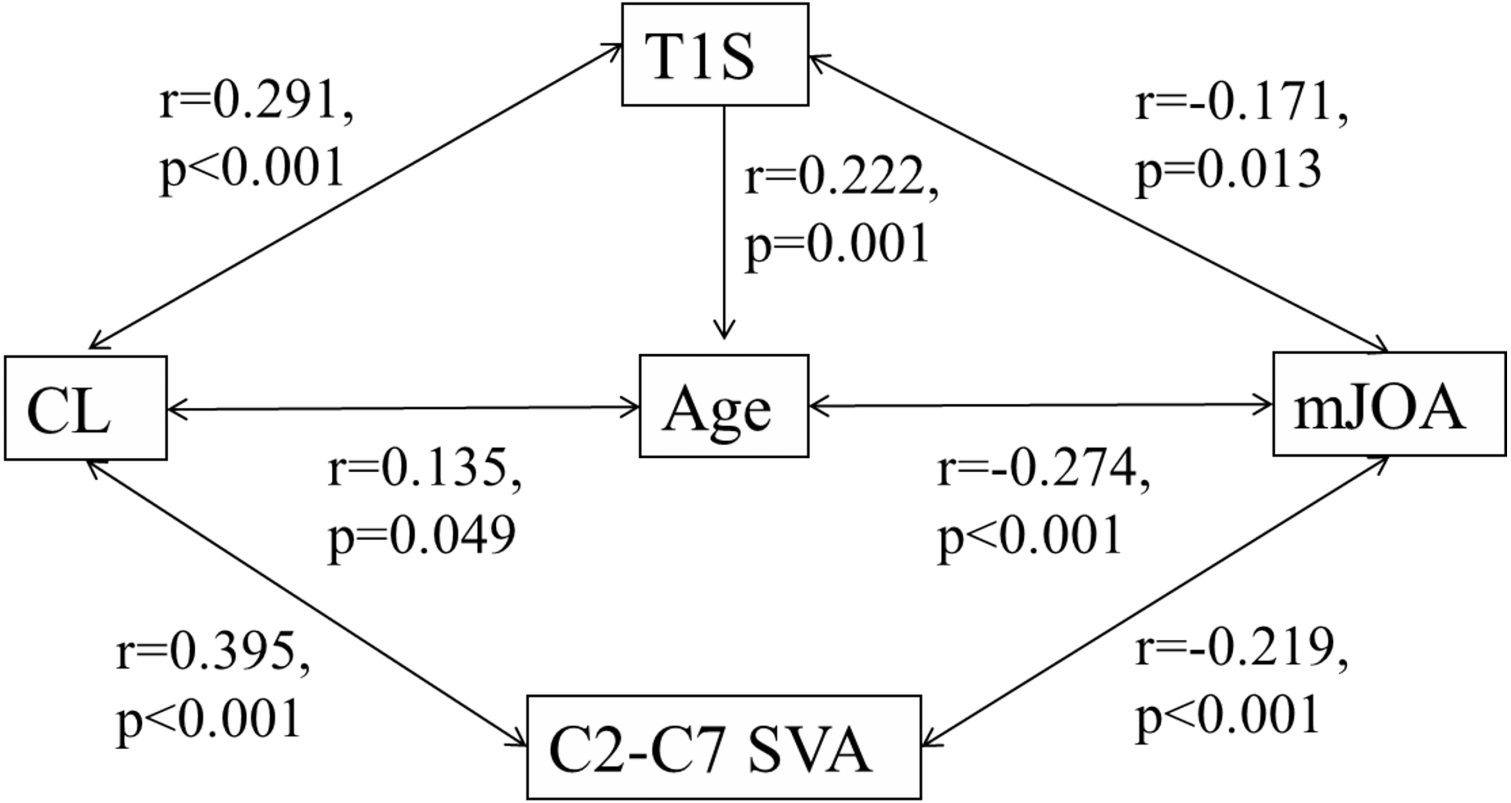
Correlation among cervical sagittal parameters.

Intraobserver and interobserver reliability for all the included radiographic parameters showed good to excellent agreement (ICC > 0.7). The details of ICC of each parameter were shown in Table 3. When examining the correlations among the parameters, we found statistically significant correlations among the following variables (Table 4): age with CL (*r* = 0.135, *P* = 0.05), age with T1S (*r* = 0.222, *P* = 0.001), CL with T1S (*r* = 0.291, *P* < 0.001), CL with C2–C7 SVA (*r* =  − 0.395, *P* < 0.001), mJOA with age (*r* =  − 0.274, *P* < 0.001), mJOA with C2–C7 SVA (*r* =  − 0.219, *P* < 0.001) and mJOA with *T*_1_*S*(*r* =  − 0.171, *P* = 0.013). The correlations of these parameters were shown in Fig. 2.

**Table 3:** Intraobserver and interobserver reliability of all radiographic parameters.

Parameters	Intraobserver ICC	Interobserver ICC
CL	0.96	0.93
C2–C7 SVA	0.98	0.97
T1S	0.96	0.95
NT	0.89	0.90
TIA	0.88	0.87

**Notes.**

CL cervical lordosis C2–C7 SVA C2–C7 sagittal vertical axis T1S T1 slope NT neck tilt TIA thoracic inlet angle ICC intraclass correlation coefficient

**Table 4:** Correlations of the parameters.

	Age	CL	C2–C7 SVA	T1S	NT	TIA
CL	0.135^*^					
C2–C7 SVA	0.076	−0.395^**^				
T1S	0.222^**^	0.291^**^	−0.001			
NT	0.125	0.007	−0.01	−0.715^**^		
TIA	0.069	0.327^**^	−0.022	0.298^**^	0.550^**^	
mJOA	−0.274^**^	0.098	−0.219^**^	−0.171^*^	0.109	−0.049

**Notes.**

* Correlation is significant at the 0.01 level (two-tailed).

** Correlation is significant at the 0.05 level (two-tailed).

CL cervical lordosis C2–C7 SVA C2–C7 sagittal vertical axis T1S T1 slope NT neck tilt TIA thoracic inlet angle mJOA modified Japanese Orthopedic Association score

Linear regression analysis on C2–C7 SVA was conducted with CL, and multiple linear regression on mJOA was conducted with age and C2–C7 SVA. Other parameters that correlated with each other were excluded to avoid multicollinearity. The results showed that CL was the predictor of C2–C7 SVA (adjusted *R*^2^ = 0.152, *P* < 0.001), and age combined with C2–C7 SVA could sensitively predict mJOA (adjusted *R*^2^ = 0.106, *P* < 0.001).

## Discussion

Degenerative changes associated with CSM can result in loss of normal sagittal alignment, and loss of CL may be the initial change of kyphosis and sagittal imbalance. Several studies suggest that cervical alignment contributes to the pathogenesis of cervical myelopathy in the CSM nature history (Ames et al., 2013a; Smith et al., 2013; Weng et al., 2016; Yoshida et al., 2017). Preoperative alignment parameters have been demonstrated to predict clinical outcomes after cervical surgery (Park et al., 2014; Tang et al., 2012). In this study, we focus on the correlations among preoperative sagittal alignment parameters and their correlations parameters with myelopathy in CSM patients. Our results showed that multiple cervical sagittal alignment parameters had internal correlations and were associated with mJOA in the preoperative patients with CSM, which would help elucidate the CSM progression.

C2–C7 SVA is an important parameter for evaluating cervical sagittal balance. According to the previous studies, the C2–C7 SVA values in asymptomatic normal volunteers are maintained in a tight range within 20 mm (Iyer et al., 2016; Jun et al., 2014; Yokoyama et al., 2017). Consistent with a previous study, the mean C2–C7 SVA value in the current study in patients with CSM was 17.2 ± 12.1 mm within the normal value, indicating that most patients with CSM before surgery presented normal sagittal balance (Mohanty et al., 2015). Moreover, there is no statistical difference between males and females in terms of sagittal alignment parameters in patients with CSM, which is in accordance with asymptomatic volunteers (Yang et al., 2016). When examining parameter correlations with C2–C7 SVA, we found that CL had positive correlations with C2–C7 SVA as reported before (Lee et al., 2015a), while T1S had no significant correlation with C2–C7 SVA. And linear regression analysis further confirmed that CL was the predictor of C2–C7 SVA. This result was different from the studies of asymptomatic normal volunteers (Knott, Mardjetko & Techy, 2010; Yang et al., 2016), which suggested that T1S was statistically correlated with C2–C7 SVA. As CL is the initial parameter affected by cervical degeneration in patients with CSM, it plays a more important role than T1S in the subsequent sagittal balance.

In terms of CL, we unexpectedly found that it had a positive correlation with age, which could be explained by the compensatory effect of CL. Firstly, T1 is the site that mainly bears the stress from head weight (Scheer et al., 2013). Long-term exposure to the stress could result in T1 tilting forward. In order to maintain a horizontal gaze, CL would increase along with the increased T1S in a compensatory manner. The increased thoracic kyphosis (TK) with aging (Iyer et al., 2016) could also account for it, as CL could compensate in a similar manner. We also found that T1S had a strong negative correlation with NT and a positive correlation with TIA. Furthermore, we observed that the formula of T1S + NT = TIA was also suitable in patients with CSM, which was firstly reported in asymptomatic normal volunteers (Lee et al., 2015b).

There is growing awareness that cervical sagittal alignment plays a pivotal role in health-related quality of life (HRQOL). An early study (Naderi et al., 1998) suggested that the presence of abnormal CL could predict limited postoperative neurological improvement. By contrast, another study assessing patients with CSM reported no significant correlation between the preoperative CL and the preoperative JOA score (Uchida et al., 2009). A double-blind, randomized controlled trial (Villavicencio et al., 2011) also suggested that improved CL did not significantly correlate with clinical outcomes. In addition, T1S has been indicated as a relevant factor on HRQOL of normal subjects (Lee et al., 2015b), and a high T1S was found to negatively affect HRQOL scores in patients with AIS (Youn et al., 2016). In patients with CSM, Smith et al. found greater myelopathy severity indicated by mJOA was correlated with worse C2–C7 SVA (Smith et al., 2013). Tang et al., (2012) suggested that C2–C7 SVA could adversely affect neck disability index (NDI) when it was beyond 40 mm. Another study concluded that increasing C2–C7 SVA was associated with greater myelopathy disability including NDI and Nurick score (Mohanty et al., 2015). The above studies suggest that CL, T1S and C2–C7 SVA are all relevant factors of HRQOL, but no consensus has been reached so far. It could be explained by the various diseases in different studies, which could lead to differences in the initial changes in the parameters and compensatory mechanisms. In the current study, mJOA was found to be significantly correlated with age, C2–C7 SVA and T1S. Also, we did not observe a correlation between CL and myelopathy severity. Only age and C2–C7 SVA were put into the multiple regression analysis model to exclude multicollinearity. The result showed that increase in age and C2–C7 SVA was significantly related to severer myelopathy. The above findings suggested that C2–C7 SVA should be corrected by surgeons to improve the clinical outcomes when treating CSM.

There are several limitations in our study. Firstly, due to the lack of global spine sagittal radiographs, the compensated changes in the lumbar spine and pelvis were not included in the current study (Ames et al., 2013b). Secondly, this study lacked of preoperative scores like NDI. In addition, the data of the control group were collected from a number of previous studies, which could contain bias in terms of region, race, etc. A further prospective study with larger study population is needed to demonstrate the dynamic changes of parameters and myelopathy in CSM.

## Conclusions

The present study showed that there were significant correlations among several preoperative cervical sagittal parameters in CSM patients. CL was the only predictor of C2–C7 SVA and age combined with C2–C7 SVA could predict the severity of myelopathy.

##  Supplemental Information

10.7717/peerj.4027/supp-1 Data S1 Raw data

## Additional Information and Declarations

### Data Availability

The following information was supplied regarding data availability: The raw data has been supplied as Data S1.
